# Finding the right help in the tumor microenvironment

**DOI:** 10.1172/JCI161052

**Published:** 2022-06-15

**Authors:** Jessica N. Filderman, Walter J. Storkus

**Affiliations:** 1 Department of Immunology,; 2 Department of Dermatology,; 3 Department of Pathology, and; 4 Department of Bioengineering, University of Pittsburgh School of Medicine, Pittsburgh, Pennsylvania, USA.; 5 UPMC Hillman Cancer Center, Pittsburgh, Pennsylvania, USA.

## Abstract

Tumor-infiltrating lymphocytes (TILs) contain substantial numbers of CD4^+^ T cells mediating pro- and antitumor functions. While CD4^+^ Tregs are well characterized and known to promote tumor immune evasion, the fingerprint of CD4^+^ Th cells that recognizes tumor antigens and serves to restrict disease progression has remained poorly discriminated. In this issue of the *JCI*, Duhen et al. analyzed tumors from patients with head and neck squamous cell carcinoma or colon carcinoma and identified a unique programmed cell death 1–positive, ICOS1-positive (PD-1^+^ICOS1^+^) subpopulation of CD4^+^ TILs highly enriched for the ability to recognize tumor-associated antigens. These cells localized proximally to MHC II^+^ antigen-presenting cells and CD8^+^ T cells within tumors, where they appeared to proliferate and function almost exclusively as Th cells. These potentially therapeutic Th cells can be monitored for patient prognosis and are expected to have substantial utility in developing personalized adoptive cell– and vaccine-based immunotherapeutic approaches for improving patient outcomes.

## An antitumor role for cognate Th cells

CD4^+^ T cells have long been appreciated to include both Th cells that facilitate antigen-specific CD8^+^ T cell and B cell responses to antigenic challenge and Tregs that suppress innate and adaptive immune responses (1–5). However, Foxp3^+^CD4^+^ Tregs have been the divas of cancer immunobiology in receiving the lion’s share of attention. Most studies dedicated to achieving a better understanding of mechanisms underlying tumor progression and optimizing immunotherapeutic gains have focused on antagonizing Foxp3^+^CD4^+^ Tregs (4, 5). Nevertheless, several recent reports have documented the phenotypic and functional heterogeneity of Th cells within the tumor microenvironment (TME) that appears to influence disease outcomes and patient responses to interventional immunotherapy (6–9). Notably, cognate CD4^+^ tumor-infiltrating lymphocytes (TILs) may directly kill MHC II^+^ tumor cells or limit tumor growth by promoting the functional conversion of protumor M2 macrophages to antitumor M1 macrophages. The transition from M2 to M1 macrophage status restricts angiogenesis and promotes the local production of cytokines and chemokines that favor proinflammatory immune cell infiltration and organization of recruited immune cells into tertiary lymphoid structures (TLSs), which have been associated with improved clinical prognosis (6–10). Therefore, it becomes important to discriminate tumor antigen–specific Th cells that persist in tumors from bystander CD4^+^ T cells that are recruited into the TME under proinflammatory conditions, but fail to be activated through their T cell receptor (TCR). While cognate CD4^+^ Th cells are responsible for mediating protective effects, bystander CD4^+^ T cells ultimately exit the tumor without providing a tangible antitumor benefit.

## Finding cognate Th cells in the TME

In this issue of the *JCI*, Duhen et al. (11) profiled the tumor immune microenvironment in patients with HPV^+^ head and neck squamous cell carcinoma (HNSCC) or microsatellite-stable colorectal carcinoma (CRC) and identified a subset of CD4^+^ Th cells coexpressing the immune checkpoint and costimulatory molecules programmed cell death 1 (PD-1) and ICOS. These double-positive (DP) cells were devoid of immune-suppressive activity and enriched in functional attributes associated with inflammatory T cell and humoral responses. Interestingly, this PD-1^+^ICOS^+^CD4^+^ T cell subpopulation exhibited a predominantly tissue-resident memory phenotype, which suggests that these cells regionally tether to tissue rather than circulate peripherally. Additionally, this cell population showed a remarkable ability to recognize oncoviral antigens (i.e., HPV-16 E6/E7 in the case of HNSCC) and mutated patient-specific tumor neoantigens (in both HNSCC and CRC) upon ex vivo activation. TCR sequencing analyses revealed that the repertoire of PD-1^+^ICOS^+^CD4^+^ TILs exhibited profound (oligo)clonal T cell expansion, with an enrichment in TCR clonotypes that were rarely found in patient-matched peripheral blood or non-DP CD4^+^ TILs. Consistent with local expansion of this CD4^+^ T cell subpopulation in the TME, and despite DP Th cell expression of the PD-1 and CTLA4 exhaustion markers, these PD-1^+^ICOS^+^ TILs appeared to be actively replicating in situ, given their coexpression of the Ki67 proliferation marker.

## A broader systems biology for tumor Th cells?

Based on tumor imaging data provided by Duhen et al., PD-1^+^ICOS^+^CD4^+^ TILs and MHC II^+^ antigen-presenting cells (APCs) were identified in close proximity within the tumor stroma (11). Such intercellular interactions may potentiate and sustain PD-1^+^ICOS^+^CD4^+^ TILs. Although Duhen and colleagues did not pedigree the Th-associated MHC II^+^ APCs by characterizing surface markers, including ICOS ligand (ICOSL) (11), B cells and macrophages express the highest levels of ICOSL within the TME (12). Since ICOS-ICOSL interactions support tissue-resident memory development (13) and T follicular helper (Tfh) survival and function (14), B cells and macrophages may constitute the principal Th-associated APCs in tumors. The finding that these Th cell–APC interactions occur in the tumor stroma, coupled with evidence for enrichment in Tfh cell content among the PD-1^+^ICOS^+^CD4^+^ TIL cohort, suggests that these lymphoid aggregates may represent one of the various maturational stages of TLSs. The presence of TLSs in the TME has generally been observed to represent a positive index for cancer prognosis and the patient’s response to interventional immunotherapies, including checkpoint blockade (10, 15).

Tfh cells and their production of CXCL13 are important for recruiting CXCR5^+^MHC II^+^ B cells into the TME and for organizing the recruited B cells into germinal centers within TLSs. Antigen-specific B cells in mature TLSs activate, proliferate, and undergo affinity maturation in the immunoglobulin repertoire before developing into antitumor, antibody-secreting plasma cells (7, 10). Physical interaction of PD-1^+^ICOS^+^ Th1 or Th17 cells with MHC II^+^ DCs in the TME would likely enhance cross-priming of antitumor CD8^+^ T cells within the tumor-associated TLSs, allowing for local antitumor T cell repertoire expansion and diversification that is distinguishable from T cell priming in the periphery. It is intriguing that Duhen et al. (11) report that the presence of stromally located DP CD4^+^ Th cells was strongly associated with CD39^+^CD103^+^CD8^+^ TIL (known to be enriched for tumor reactivity) content in the TME of HNSCC samples, but did not find such an association in CRC specimens. Differences in TME content of regulatory immune cells (i.e., Foxp3^+^CD4^+^ Tregs and myeloid-derived suppressor cells) or tumor-intrinsic immune suppression mechanisms were not accounted for in these analyses and could therefore serve as potential mechanisms underlying the divergence in the immune cell interactions operating in the TME of HNSCC compared with that of CRC.

## Translational ramifications of these findings

**Figure 1:**
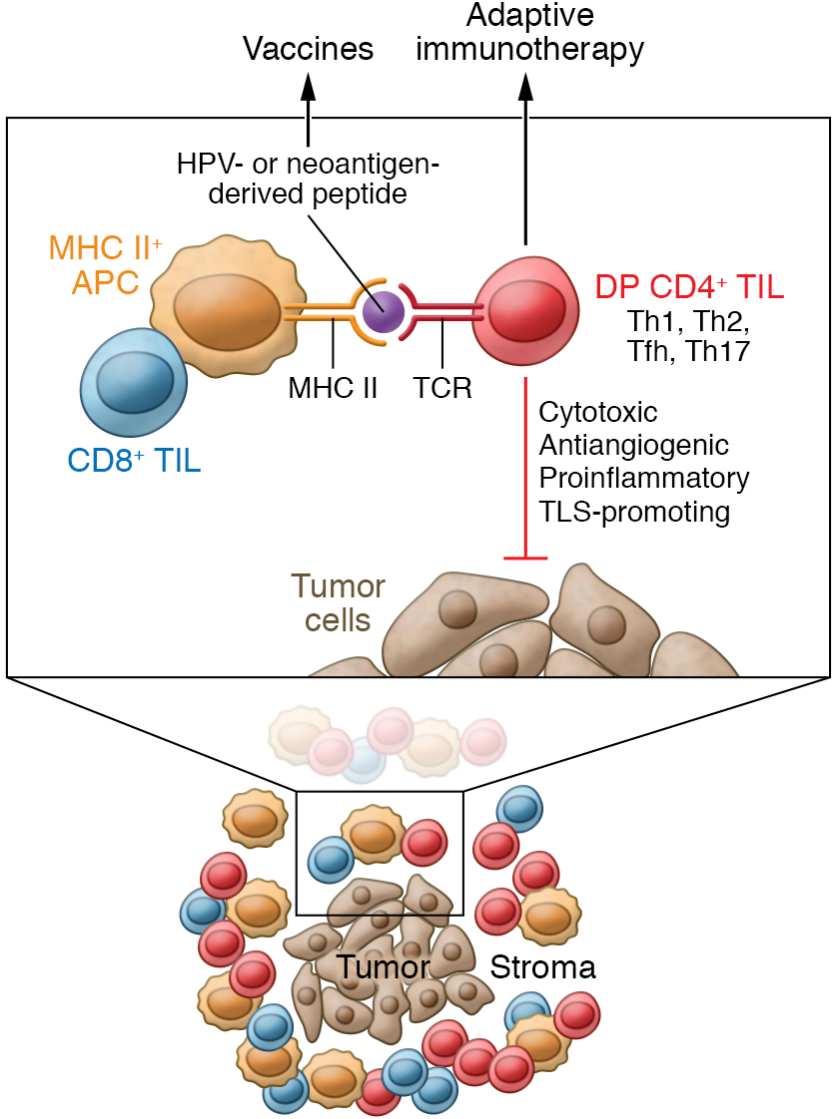
PD-1^+^ICOS^+^CD4^+^ TILs in HNSCC and CRC tumors are enriched for the tissue-resident memory cell phenotype and reactivity to tumor antigens. Tumor-derived DP Th cells recognize cancer-relevant antigens, maintain polyfunctionality, and mediate primarily non-Treg functions in the TME. In the study by Duhen et al. (11), DP cells represented a substantial proportion of CD4^+^ TILs in HNSCC and CRC tumor specimens and were enriched in HPV-reactive Th cells (from HNSCC samples) and neoantigen-reactive Th cells (from HNSCC and CRC samples). Tumor-reactive Th cells included several functional subsets: Th1 (marked by *TBET*, *IFNG*), Th2 (marked by *GATA3*, *IL13*), Tfh (marked by *BCL6*, *CXCL13*, *IL21*), and Th17 (marked by *RORC*, *IL17A*). Within the stroma of the TME, DP CD4^+^ TILs were organized in lymphoid aggregates with MHC II^+^ APCs and CD8^+^ T cells reminiscent of immature TLSs. A DP CD4^+^ TIL status may provide a useful index for patients’ prognosis and response to interventional immunotherapy, with the isolated cells and targeted antigens serving as the basis for personalized adoptive cell therapies and patient-specific vaccines, respectively.

So how is this information instructive for improving response rates to interventional immunotherapies in the setting of HNSCC and CRC or other forms of solid cancer? As Duhen and authors note, their findings suggest the possibility of sorting functional DP CD4^+^ TILs from tumor biopsy material to provide an enriched source of nonexhausted, Treg-depleted, tumor antigen–specific T cells that could be expanded ex vivo for subsequent transfer back into the patient as an adoptive immunotherapy (11, 16, 17). Alternatively, TCRs cloned from DP CD4^+^ TILs reactive against patient-matched tumor (neo)antigens could be used to develop TCR-engineered T cells from the patient’s peripheral blood for adoptive transfer as an interventional therapy (16, 17). In addition, tumor-associated peptide epitopes, such as those derived from patients’ neoantigens identified by exome sequencing and in vitro T cell reactivity screening, could be applied in vaccine formulations (18, 19) to activate the patient’s peripheral CD4^+^ Th cell repertoire or to expand and support in vivo maintenance of adoptively transferred T cells reactive against these MHC II–presented peptides (Figure 1). To bolster the in situ antitumor activity of tumor-resident PD-1^+^ICOS^+^CTLA4^+^CD4^+^ TILs, one could obviously also contemplate treating patients with cancer with anti–PD-1/anti-CTLA4 antagonist antibodies and/or anti-ICOS agonist antibodies. The use of anti-ICOS antibodies would have conceptual limitations, however, since ICOS is expressed by Tregs and some tumor cells. Therefore, systemic agonism of ICOS-mediated signaling in these cells could also result in protumor effects (20). Further transcriptional profiling of DP CD4^+^ TILs may uncover additional biomarkers and pathway factors that may be therapeutically targeted to selectively reinforce or improve tumor antigen–specific DP Th cell bioactivity within the TME to benefit patient outcomes.

## Version 1 06/15/2022

Electronic publication
